# Association between ethylene oxide exposure and periodontitis: a cross-sectional study from NHANES 2013–2014

**DOI:** 10.1186/s12889-024-17735-3

**Published:** 2024-01-16

**Authors:** Di Miao, Lina Zhao, Ruoyan Cao

**Affiliations:** 1 Key Laboratory of Shaanxi Province for Craniofacial Precision Medicine Research, College of Stomatology, Xi’an Jiaotong University, Xi’an, China; 2 Department of Periodontology, College of Stomatology, Xi’an Jiaotong University, Xi’an, China; 3 Department of Stomatology, Tongde Hospital of Zhejiang Province, Hangzhou, China; 4 Department of Periodontics, Liaoning Provincial Key Laboratory of Oral Diseases, School and Hospital of Stomatology, China Medical University, Nanjing North Street 117, Shenyang, Liaoning 110002 China

**Keywords:** Periodontitis, Ethylene oxide, NHANES, Cross-sectional study

## Abstract

**Background:**

Exposure to ethylene oxide (EO) induces inflammation and oxidative stress, which are the main mechanisms of periodontitis. However, the effect of EO on periodontal health is not unclear. In this study, we aimed to explore the relationship between EO exposure and the risk of periodontitis in general US adults.

**Methods:**

Data used in our study from the National Health and Nutritional Examination Survey (NHANES) 2013–2014. The EO biomarker, hemoglobin adduct of EO (HbEO), was measured in blood samples utilizing high-performance liquid chromatography-tandem mass spectrometry. Periodontitis category was defined by the CDC/AAP according to clinical periodontal parameters. Natural cubic spline, weight multivariable logistic regression analyses and subgroup analysis were used to explore the association between EO exposure and the risk of periodontitis.

**Results:**

A total of 1497 participants over the age of 30 were included in our study. A non-linear positive association with periodontitis was identified for HbEO levels. Participants in the highest tertile of HbEO levels were more likely to have poorer periodontal health compared to the lowest tertile (OR_tertile3vs1_ = 2.80, 95% CI: 1.85–4.24). Similar results were also found in different subgroups.

**Conclusions:**

HbEO levels are positively associated with poor periodontal health in US adults. Additional longitudinal studies are necessary to further enhance our comprehension of the impact of exposure to EO on periodontal status.

**Supplementary Information:**

The online version contains supplementary material available at 10.1186/s12889-024-17735-3.

## Background

Periodontitis is the most common inflammatory disease caused by microorganisms, resulting in loss of attachment and resorption of alveolar bone, ultimately leading to tooth loss [1]. An estimated 42% of US adults over the age of 30 suffer from periodontal disease, which affects oral health and life quality [2]. Periodontitis is associated with a variety of systemic diseases, such as cardiovascular disease [3]. Growing evidence suggests that environmental pollutants may be involved in the occurrence and development of cardiovascular disease [4–6], however, less evidence exists between environmental pollutants and periodontitis.

Ethylene oxide (EO) is an industrial chemical primarily utilized as a sterilant for medical devices and as an intermediate in the production of other chemicals [7]. EO exists in gaseous at room temperature, therefore the main route of exposure is through inhalation. Exposure to EO in the general population occurs mainly through contaminated air, cigarette smoke and vehicle exhaust fumes [8]. In addition, EO sterilization is responsible for sterilizing a significant portion, around 50%, of all sterile medical devices within the United States. Therefore, with the prevalence of COVID-19 and the increased demand for personal protective equipment such as masks, gloves, and gowns, there is likely to be a rise in exposure to EO as well [6]. EO is linked to inflammation and oxidative stress, which are also major mechanism contributing to periodontitis [9, 10]. Given the such relationship, we hypothesized that EO was positively associated with poor periodontal health.

EO is a widely recognized alkylating agent that reacts with valine in hemoglobin. Hemoglobin adducts of EO (HbEO) are highly effective and sensitive biomarkers for evaluating exposure to EO. In this study, we aimed to assess the independent relationship between EO exposure as measured by blood HbEO levels, and periodontitis based on the data from the National Health and Nutrition Examination Survey (NHANES). We also explored this association in different subgroups.

## Methods

### Study population

The data used in this study were downloaded from the NHANES 2013–2014 [4, 11]. A representative population sample was acquired based on a cluster, stratified, multistage sampling method and cross-sectional study design. NHANES participants are chosen on an annual basis by considering various factors such as counties, blocks, households, and individuals residing within households. To ensure accurate estimation, certain groups, including Mexican Americans and Non-Hispanic Blacks, are intentionally oversampled. The National Center for Health Statistics, which is affiliated with the Centers for Disease Control and Prevention, approved the NHANES protocol, and all participants signed an informed consent form. Individuals without complete full-mouth periodontal examination (FMPE), missing HbEO levels, and participants younger than 30 years were excluded. Finally, a total of 1497 participants were included in our study.

### Exposure variable

The washed, packed red blood cell samples underwent proper processing, storage, and shipment to the Division of Laboratory Sciences for thorough analysis. The main objective was to determine the total level of hemoglobin in order to assess the presence of hemoglobin adducts. To achieve this, the reaction mixture was carefully combined with the sample, followed by the isolation of Edman degradation products. Subsequently, the products were identified and detected using high-performance liquid chromatography coupled with tandem mass spectrometry (HPLC-MS/MS). Results were quantified and expressed as pmol adducts per gram of hemoglobin. The detection of hemoglobin was carried out using a well-established, commercially available assay kit. Blood HbEO is expressed in pmol/g hemoglobin with a detection limit of 12.90 pmol/g hemoglobin.

### Outcome variable

The outcome of this study was moderate or severe periodontitis. The dental examiners underwent an extensive period of training and calibration to ensure the accuracy and quality of periodontal health data. This process included continuous monitoring and recalibration to maintain the required standards [12]. Periodontal examination contained attachment loss (AL) and probing pocket depth (PPD) at six sites per tooth without third molars based on the FMPE protocol. A maximum of 168 sites and 28 teeth per subject could be examined to assess periodontal status. CDC/AAP definitions were used for the classification of periodontitis [13]. No/mild periodontitis was characterized as no evidence of moderate/severe periodontitis; moderate periodontitis: ≥ 2 interproximal sites with PD ≥ 5 mm not on the same tooth, or ≥ 2 interproximal sites with CAL ≥ 4 mm not on the same tooth; severe periodontitis: ≥ 2 interproximal sites with CAL ≥ 6 mm not on the same tooth and ≥ 1 interproximal sites with PD ≥ 5 mm.

### Covariates

Covariates were collected from previous studies, including age, gender, race, education level, poverty index (PI), marital status, smoking status, alcohol consumption, obesity, diabetes mellitus and hypertension. The detail of covariates collect could obtain from the NHANES database [14]. We classified race into two categories: non-Hispanic white and others. Education level was categorized as follows: < high school (less than 9th grade or 9th to 11th grade), high school (high school grade/GED or equivalent), and > high school (some college/AA degree or college graduate or above). The poverty index (PI) was determined by calculating the ratio of family income to the poverty level, as defined by the Department of Health and Human Services poverty guidelines. We categorized PI into three groups: ≤ 1.3, 1.3–3.5, and > 3.5. Marital status was grouped as follows: married/living as married, never married, and separated/divorced/widowed. Smoking status was divided based on whether individuals had smoked less than 100 cigarettes in their lifetime or not. Alcohol consumption was grouped based on whether individuals had consumed at least 12 drinks or not [15]. Body mass index (BMI) was calculated by dividing weight (in kilograms) by the square of height (in meters, m^2). Obesity was defined as a BMI ≥ 30 kg/m^2, while a BMI < 30 kg/m^2 was classified as non-obesity [16]. The diagnosis of diabetes mellitus was based on self-reported physician diagnosis or a glycosylated hemoglobin level (HbA1c) ≥ 6.5% or a fasting blood glucose ≥ 7.0 mmol/L or current use of hypoglycemic drugs. The diagnosis of hypertension was based on self-reported physician diagnosis or current use of antihypertensive medications or a systolic blood pressure ≥ 130 mmHg or a diastolic blood pressure ≥ 80 mmHg [17].

### Statistical analysis

Weights were considered in our study based on the NHANES analysis guide. Categorical variables were presented as percentage. Baseline characteristics were compared across the tertiles of HbEO levels using Chi-square test for categorical variables. We performed log2-transformation of HbEO levels owing to the skewed distribution found [18]. Natural cubic splines was used to identify any non-linear relationship of HbEO levels with moderate/severe periodontitis. We evaluated the relationship between HbEO levels and periodontitis using weighted multivariable logistic regression models. The analysis was performed using the ‘svyglm’ function with family = binomial in the R software. The outcome variable of this study was periodontitis, which was categorized into two groups: no/mild, and moderate/heavy. The exposure variable was the grouping of HbEO levels into tertiles. Model I was adjusted for age, gender and race, and model II was adjusted for age, gender, race, education level, marital status, PI, obesity, smoking status, alcohol consumption, diabetes and hypertension. In addition, we performed stratified and interaction analyses to assess whether the association between the HbEO levels tertiles and periodontitis differed by all variables in Table 1. We created a separate category for covariates with missing observations and conducted regression analysis to account for missing observations and their potential effect on the outcome [19]. All the analyses were performed using R software (version 4.1.2). A *P*-value less than 0.05 was considered significant.

**Table 1:** Weighted characteristics of the participates

Characteristics	HbEO levels				
	Overall n = 1497	Tertile 1 n = 501	Tertile 2 n = 497	Tertile 3 n = 499	*P*-value
Age group (%)					< 0.001
≤ 60 years	77.14	72.56	71.23	88.6	
> 60 years	22.86	27.44	28.77	11.4	
Gender (%)					0.24
Female	49.29	51.76	46.72	48.66	
Male	50.71	48.24	53.28	51.34	
Race (%)					0.07
Non-Hispanic White	68.58	71.98	62.02	70.61	
Others	31.42	28.02	37.98	29.39	
Marital status (%)					< 0.001
Married/living as married	65.51	73.02	67.02	54.55	
Never married	13.09	10.27	11.22	18.45	
Separated/divorced/widowed	21.4	16.7	21.76	27	
Education level (%)					< 0.0001
< High school	14.66	9.52	15.32	20.51	
High school	24.26	17.55	22.03	34.86	
> High school	61.09	72.92	62.64	44.63	
PI (%)^a^					< 0.0001
≤ 1.3	22.77	11.94	21.8	37.4	
1.3–3.5	32.93	29.85	30.49	39.18	
> 3.5	38.92	52.86	42.14	18.21	
Obesity (%)^a^	39.12	41.31	38.47	36.98	0.51
Smoker (%)	56.76	28.57	51.06	97.88	< 0.0001
Alcohol consumption (%)^a^	78.17	75.77	76.01	83.28	0.01
Diabetes mellitus (%)^a^	9.9	8.84	13.73	7.55	< 0.001
Hypertension (%)	42.05	41.49	41.07	43.7	0.82
Periodontitis (%)					< 0.0001
Non/mild periodontitis	61.52	75.05	63.31	42.68	
Moderate/severe periodontitis	38.48	24.95	36.69	57.32	

^a^Missing values for total study: PI (5.38%), obesity (0.21%), alcohol consumption (5.81%), and diabetes mellitus (0.33%)

## Results

### Baseline characteristics

**Fig. 1:**
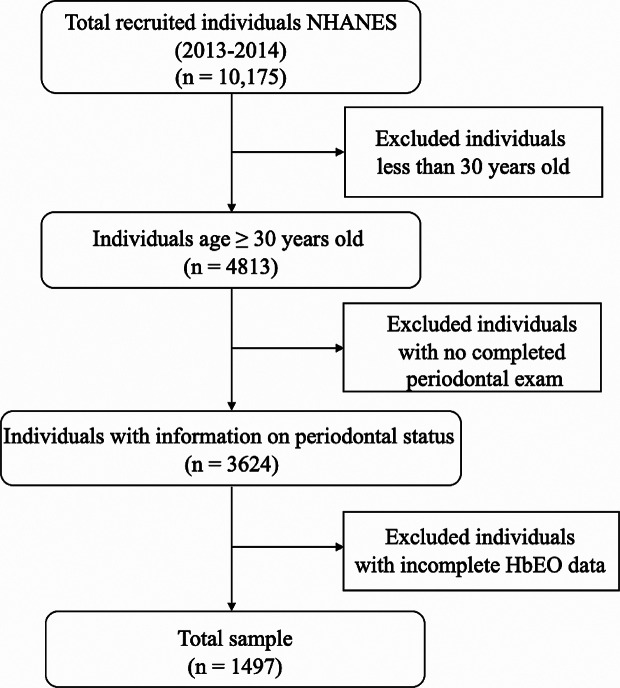
Study population selection

As shown in Fig. 1, this cross-section study included a total of 1497 NHANES participants, which represented approximately 61.3 million noninstitutionalized residents of the United States. The baseline characteristics of our study in the tertiles of HbEO levels were presented in Table 1. The prevalence of gender, race, obesity, and hypertension was similar among the three different HbEO levels. There were significant differences in age, marital status, education level, PI, smoking status, alcohol consumption, the prevalence of diabetes mellitus and periodontitis among different HbEO levels. The prevalence of moderate/severe periodontitis across the tertiles of HbEO levels was 24.95%, 36.69% and 57.32%, respectively.

### Associations between blood EO levels and periodontitis

**Fig. 2:**
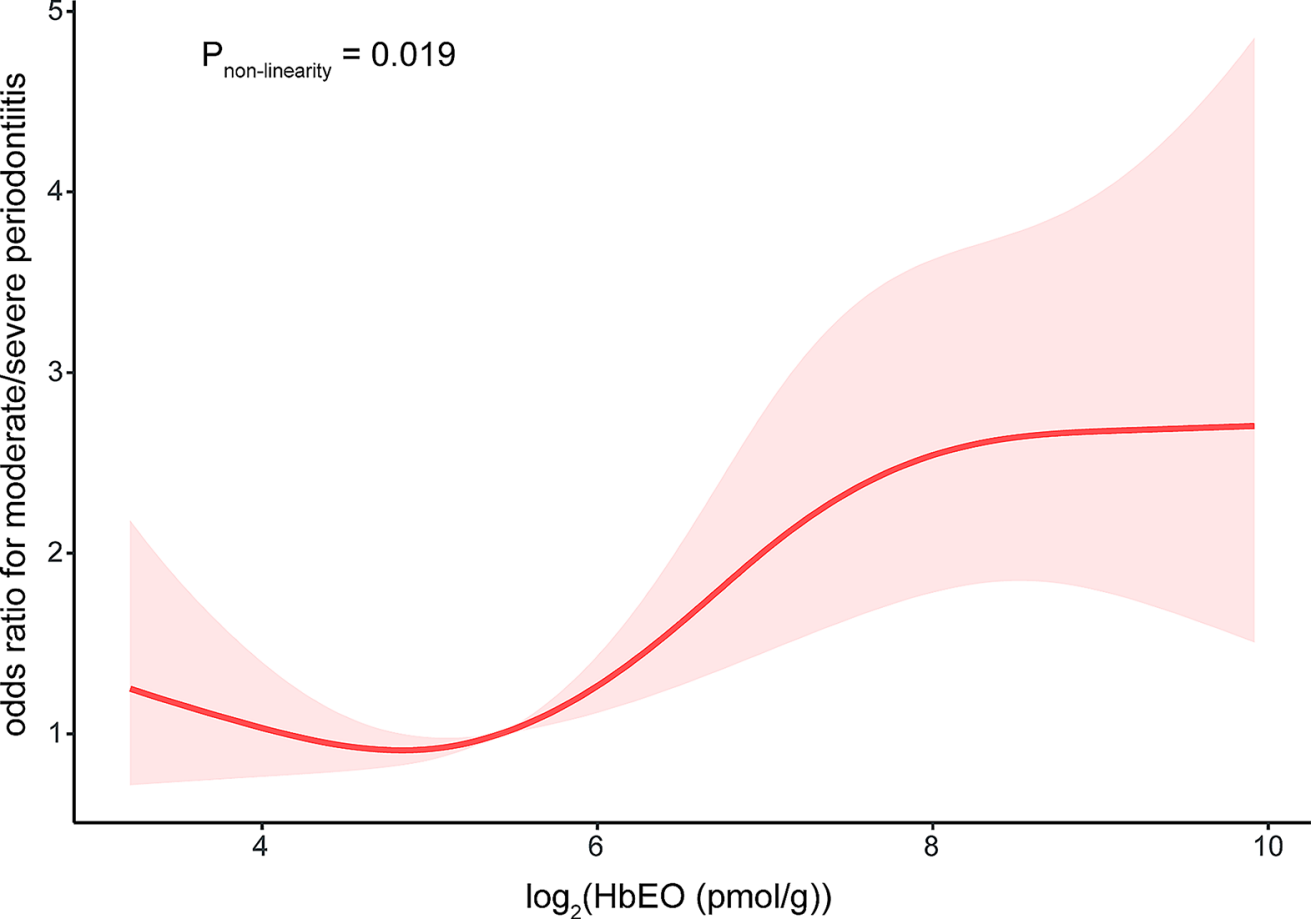
The relationship between EO exposure and the risk of moderate/severe periodontitis

Figure 2 showed a non-linear positive correlation between HbEO levels and poor periodontal health. Therefore, we grouped HbEO levels into tertiles for further analysis. The odds ratios (OR) with 95% confidence intervals (CIs) of periodontal disease based on the tertiles of HbEO levels were shown in Table 2. High level of HbEO was positively associated with moderate/severe periodontitis in the different models: Crude model (OR_tertile3vs1_ = 4.04, 95% CI: 2.84–5.74, Model I (OR_tertile3vs1_ = 5.24, 95% CI: 5.24–7.72 and Model II (OR_tertile3vs1_ = 2.80, 95% CI: 1.85–4.24).

**Table 2:** Association between blood HbEO levels and periodontitis

Variable	Crude model			Model I			Model II	
	OR (95%CI)	*P*-value		OR (95%CI)	*P*-value		OR (95%CI)	*P*-value
EO exposure								
Tertile 1	Ref			Ref			Ref	
Tertile 2	1.74 (1.09, 2.80)	0.02		1.62 (0.95, 2.78)	0.07		1.33 (0.86, 2.07)	0.19
Tertile 3	4.04 (2.84, 5.74)	< 0.0001		5.24 (3.55, 7.72)	< 0.0001		2.80 (1.85, 4.24)	< 0.0001
*P* for trend	< 0.0001			< 0.0001			< 0.0001	

Model I: Adjusted for age, gender and race

Model II: Model I and adjusted for education level, marital status, PI, obesity, smoking status, alcohol consumption, diabetes mellitus and hypertension

### Subgroup analysis

The subgroup analyses on the relationship between HbEO levels and moderate/severe periodontitis were shown in Table S1. High levels of HbEO were found to be positively correlated with moderate/severe periodontitis in most subgroups, with the exception of individuals who had never been married, had smoked fewer than 100 cigarettes in their lifetime, were obese, or had diabetes mellitus. However, no significant interactions were observed for any of the variables presented in Table 1.

## Discussion

In this cross-sectional study, we found that HbEO levels were positively associated with moderate/severe periodontitis in the US adult population. Additionally, this association remained significant in most subgroups. Our study provides evidence linking EO exposure with periodontitis.

To minimize potential confounding, we employed multivariable logistic regression to adjust for various important covariates such as age, gender, race, education level, marital status, socioeconomic status, obesity, smoking status, alcohol consumption, diabetes mellitus, and hypertension. The results also revealed a significant association between HbEO levels and periodontitis. One significant advantage of using multivariable regression methods is their ability to incorporate data from all individuals included in the study. Furthermore, this technique is widely acknowledged and comprehensible among researchers, making the analysis easily attainable through commonly used software applications [20]. Additionally, including additional variables in the adjusted models could potentially improve the model fit and enhance precision. However, it is important to note that this approach only takes into account the influence of covariates that were specifically measured. Additionally, it may lead to overfitting, collinearity issues, and necessitate a larger sample size to maintain sufficient statistical power. Moreover, it can impact the generalizability of the results by narrowing the scope of the study population.

EO is commonly regarded as a primary hazard for workers in sterilization facilities, particularly those involved in the sterilization of medical devices. Additionally, attention should be drawn to the potential risk of EO exposure in the general population, which can result from volatile organic compound exposure through renovation activities, smoking, and residing close to facilities that utilize ethylene oxide [18]. Exposure to EO could lead to a range of adverse health effects, including cancer, diabetes mellitus, cardiovascular disease and hypertension [5, 8, 21, 22]. These adverse health effects are also associated with periodontitis. Thus, it is reasonable for us to speculate that EO exposure might be linked to periodontal health. As a result, we found that HbEO levels was positively associated with moderate/severe periodontitis. Additionally, we observed a consistent trend across different subgroups, which indicates the strength and reliability of the association between exposure to EO and periodontitis.

EO-induced inflammation and oxidative stress might contribute to accelerated periodontal tissue destruction [9, 10]. EO levels are positively related to high-sensitivity C-reactive protein (hs-CRP) and alkaline phosphatase (ALP) [6]. It is well known that hs-CRP, an inflammatory marker, is elevated in patients with periodontitis and decreased after periodontal therapy [23]. Recent evidence indicates that CRP involves in the regulation of alveolar bone homeostasis in periodontitis. CRP knockout reduces the alveolar bone loss and osteoclastogenic markers expression, while increasing osteogenic markers expression in vivo [24]. ALP is required for bone mineralization [25], and thus decreased expression of ALP is linked to bone loss.

Evidence indicates that long-term chronic exposure to EO could reduce the activity of glutathione reductase, which is correlated with increasing reactive oxygen species (ROS) [26]. ROS-induced oxidative stress is a hallmark of periodontitis and plays an important role in the destruction of periodontium. Oxidative stress may promote the production of proinflammatory cytokines and chemokines via the activation of NLRP3-, NF-κB-, JNK- and dependent pathways [27]. Additionally, matrix metalloproteinase (MMP), such as MMP2, MMP8, MMP-9 and MMP13, could be activated by oxidative stress [28, 29]. MMPs are involved in enhancing extracellular matrix degradation and prolonging inflammation [30].

This study has several limitations that should be noted. Firstly, it is unfeasible to reflect the causal relationship between HbEO levels and poor periodontal health based on a cross-sectional design. Secondly, a single measurement of HbEO levels was used in this study, while dynamic changes in HbEO levels may result in exposure misclassification. Thirdly, the contribution of cigarette smoke to exposure to EO makes passive smokers an important subgroup. However, the NHANES database does not provide any information to identify individuals who are passive smokers. Finally, we could not rule out all possible residual confounders due to unmeasured confounding factors.

## Conclusions

In summary, HbEO levels is positively associated with poor periodontal health among US adults. It is necessary for individuals who are exposed to higher than average levels of EO in their environment to prioritize improving their oral hygiene and promoting regular periodontal examinations. Further prospective trials are also necessary to corroborate our findings, and additional investigations are required to delve deeper into the related mechanisms.

### Electronic supplementary material

Below is the link to the electronic supplementary material.

Supplementary Material 1

## Data Availability

The NHANES data of our study are openly available at https://www.cdc.gov/nchs/nhanes/default.aspx.

## Abbreviations

NHANES: National Health and Nutrition Examination Survey
EO: Ethylene oxide
HbEO: Hemoglobin adduct of ethylene oxide
FMPE: Full-mouth periodontal examination
AL: Attachment loss
PPD: Probing pocket depth
PI: Poverty index
SD: Standard deviation
OR: Odds ratios
CIs: Confidence intervals
hs-CRP: High-sensitivity C-reactive protein
ALP: Alkaline phosphatase
ROS: Reactive oxygen species

## References

[1] Papapanou PN, Sanz M, Buduneli N, Dietrich T, Feres M, Fine DH, Flemmig TF, Garcia R, Giannobile WV, Graziani F (2018). Periodontitis: Consensus report of workgroup 2 of the 2017 World workshop on the classification of Periodontal and Peri-implant diseases and conditions. J Periodontol.

[2] Eke PI, Thornton-Evans GO, Wei L, Borgnakke WS, Dye BA, Genco RJ. Periodontitis in US Adults: National Health and Nutrition Examination Survey 2009–2014. *Journal of the American Dental Association (*1939*)* 2018, 149(7):576–588.e576. 10.1016/j.adaj.2018.04.023 PMC8094373 29957185

[3] Sabharwal A, Gomes-Filho IS, Stellrecht E, Scannapieco FA (2018). Role of periodontal therapy in management of common complex systemic diseases and conditions: an update. Periodontol 2000.

[4] Glover F, Eisenberg ML, Belladelli F, Del Giudice F, Chen T, Mulloy E, Caudle WM (2022). The association between organophosphate insecticides and blood pressure dysregulation: NHANES 2013–2014. Environ Health: Global Access Sci Source.

[5] Wu N, Cao W, Wang Y, Liu X. Association between blood ethylene oxide levels and the prevalence of hypertension. Environ Sci Pollut Res Int 2022. 10.1007/s11356-022-21130-z 35668269

[6] Zhu X, Kong X, Chen M, Shi S, Cheang I, Zhu Q, Lu X, Yue X, Tang Y, Liao S (2022). Blood ethylene oxide, systemic inflammation, and serum lipid profiles: results from NHANES 2013–2016. Chemosphere.

[7] Shintani H (2017). Ethylene Oxide Gas sterilization of Medical devices. Biocontrol Sci.

[8] Guo J, Wan Z, Cui G, Pan A, Liu G (2021). Association of exposure to ethylene oxide with risk of diabetes mellitus: results from NHANES 2013–2016. Environ Sci Pollut Res Int.

[9] Rasool M, Malik A, Abdul Basit Ashraf M, Mubbin R, Ayyaz U, Waquar S, Asif M, Umar M, Siew Hua G, Iqbal Z (2021). Phytochemical analysis and protective effects of Vaccinium macrocarpon (cranberry) in rats (Rattus norvegicus) following ethylene oxide-induced oxidative insult. Bioengineered.

[10] Adedara IA, Farombi EO (2010). Induction of oxidative damage in the testes and spermatozoa and hematotoxicity in rats exposed to multiple doses of ethylene glycol monoethyl ether. Hum Exp Toxicol.

[11] Fain JA (2017). NHANES Diabetes Educ.

[12] Dye BA, Afful J, Thornton-Evans G, Iafolla T (2019). Overview and quality assurance for the oral health component of the National Health and Nutrition Examination Survey (NHANES), 2011–2014. BMC Oral Health.

[13] Eke PI, Page RC, Wei L, Thornton-Evans G, Genco RJ (2012). Update of the case definitions for population-based surveillance of periodontitis. J Periodontol.

[14] [https://wwwn.cdc.gov/nchs/nhanes/continuousnhanes/default.aspx?BeginYear=2013].

[15] Cheang I, Zhu X, Zhu Q, Li M, Liao S, Zuo Z, Yao W, Zhou Y, Zhang H, Li X (2022). Inverse association between blood ethylene oxide levels and obesity in the general population: NHANES 2013–2016. Front Endocrinol (Lausanne).

[16] Costa SA, Ribeiro CCC, Moreira ARO, Carvalho Souza SF (2022). High serum iron markers are associated with periodontitis in post-menopausal women: a population-based study (NHANES III). J Clin Periodontol.

[17] Chen H, Zhang X, Luo J, Dong X, Jiang X (2022). The association between periodontitis and lung function: results from the National Health and Nutrition Examination Survey 2009 to 2012. J Periodontol.

[18] Han L, Wang Q (2023). Association between hemoglobin adducts of ethylene oxide levels and the risk of short sleep duration in the general population: an analysis based on the National Health and Nutrition Examination Survey. Environ Sci Pollut Res Int.

[19] Shi L, Zhu Z, Tian Q, He L. Association of Interdental Cleaning and Untreated Root Caries in adults in the United States of America. Int Dent J 2023. 10.1016/j.identj.2023.04.004 PMC10658437 37316412

[20] Normand SL, Sykora K, Li P, Mamdani M, Rochon PA, Anderson GM (2005). Readers guide to critical appraisal of cohort studies: 3. Analytical strategies to reduce confounding. BMJ.

[21] Jinot J, Fritz JM, Vulimiri SV, Keshava N (2018). Carcinogenicity of ethylene oxide: key findings and scientific issues. Toxicol Mech Methods.

[22] Zeng G, Zhang Q, Wang X, Wu KH (2021). Association between blood ethylene oxide levels and the risk of cardiovascular diseases in the general population. Environ Sci Pollut Res Int.

[23] Luthra S, Orlandi M, Hussain SB, Leira Y, Botelho J, Machado V, Mendes JJ, Marletta D, Harden S, D’Aiuto F. Treatment of Periodontitis and C-Reactive protein: a systematic review and Meta-analysis of Randomized clinical trials. J Clin Periodontol 2022. 10.1111/jcpe.13709 PMC10087558 35946825

[24] Zhou M, Xu X, Li J, Zhou J, He Y, Chen Z, Liu S, Chen D, Li H, Li G et al. C-reactive protein perturbs alveolar bone homeostasis: an experimental study of periodontitis and diabetes in the rat. J Clin Periodontol 2022. 10.1111/jcpe.13667 35634690

[25] Liu W, Zhang L, Xuan K, Hu C, Li L, Zhang Y, Jin F, Jin Y (2018). Alkaline phosphatase controls lineage switching of mesenchymal stem cells by regulating the LRP6/GSK3β complex in Hypophosphatasia. Theranostics.

[26] Katoh T, Higashi K, Inoue N, Tanaka I (1988). Effects of chronic inhalation of ethylene oxide on lipid peroxidation and glutathione redox cycle in rat liver. Res Commun Chem Pathol Pharmacol.

[27] Vo TTT, Chu PM, Tuan VP, Te JS, Lee IT. The promising role of antioxidant phytochemicals in the Prevention and Treatment of Periodontal Disease via the inhibition of oxidative stress pathways: updated insights. Antioxid (Basel Switzerland) 2020, 9(12). 10.3390/antiox9121211 PMC7760335 33271934

[28] Osorio C, Cavalla F, Paula-Lima A, Díaz-Araya G, Vernal R, Ahumada P, Gamonal J, Hernández M (2015). H2 O2 activates matrix metalloproteinases through the nuclear factor kappa B pathway and ca(2+) signals in human periodontal fibroblasts. J Periodontal Res.

[29] Hernández-Ríos P, Pussinen PJ, Vernal R, Hernández M (2017). Oxidative stress in the local and systemic events of apical periodontitis. Front Physiol.

[30] Wan CY, Li L, Liu LS, Jiang CM, Zhang HZ, Wang JX (2021). Expression of Matrix metalloproteinases and tissue inhibitor of Matrix metalloproteinases during apical Periodontitis Development. J Endod.

