# Supplementary material for: Association between ethylene oxide exposure and periodontitis: a cross-sectional study from NHANES 2013–2014
Source: BMC Public Health. 2024 Jan 16;24:195. doi: 10.1186/s12889-024-17735-3 (PMC10790520; doi:10.1186/s12889-024-17735-3)
Supplement: Supplementary file 1 — Supplementary Material 1 [file 12889_2024_17735_MOESM1_ESM.docx]

*Table S1* Effect size of blood HbEO levels on periodontitis in each subgroup

| Characteristic | EO exposure | | | *P* for trend | *P* for interaction |
| --- | --- | --- | --- | --- | --- |
|  | Tertile 1 | Tertile 2 | Tertile 3 |  |  |
| Age |  |  |  |  | 0.36 |
| ≤ 60 | Ref | 0.98 (0.62, 1.53) | **2.13 (1.25, 3.63)** | 0.01 |  |
| > 60 | Ref | 2.06 (0.85, 4.97) | **5.44 (1.30, 22.70)** | 0.12 |  |
| Gender |  |  |  |  | 0.55 |
| Female | Ref | 1.27 (0.76, 2.13) | **3.20 (1.79, 5.71)** | 0.52 |  |
| Male | Ref | 1.53 (0.72, 3.26) | **2.47 (1.19, 5.15)** | 0.44 |  |
| Race |  |  |  |  | 0.27 |
| Non-Hispanic White | Ref | 1.51 (0.78, 2.93) | **2.44 (1.38, 4.33)** | 0.27 |  |
| Others | Ref | 1.07 (0.66, 1.72) | **3.68 (2.31, 5.87)** | 0.72 |  |
| Education level |  |  |  |  | 0.94 |
| < High school | Ref | 1.19 (0.61, 2.32) | **3.84 (1.23, 11.98)** | 0.79 |  |
| High school | Ref | 1.40 (0.83, 2.37) | **2.59 (1.45, 4.62)** | 0.005 |  |
| > High school | Ref | 1.41 (0.78, 2.54) | **3.04 (1.80, 5.13)** | 0.28 |  |
| PI |  |  |  |  | 0.28 |
| ≤ 1.3 | Ref | **2.28 (1.15, 4.50)** | **2.96 (1.28, 6.88)** | 0.02 |  |
| 1.3-3.5 | Ref | 1.06 (0.51, 2.20) | **2.33 (1.03, 5.29)** | 0.12 |  |
| > 3.5 | Ref | **1.04 (0.39, 2.81)** | **2.99 (1.24, 7.18)** | 0.04 |  |
| Marital status |  |  |  |  |  |
| Married/living as married | Ref | 1.20 (0.62, 2.32) | **2.84 (1.37, 5.92)** | 0.01 | 0.58 |
| Never married | Ref | 1.39 (0.48, 4.02) | 1.50 (0.39, 5.70) | 0.55 |  |
| Separated/divorced/widowed | Ref | **2.39 (1.25, 4.58)** | **6.21 (2.56, 15.06)** | 0.07 |  |
| Smoking status |  |  |  |  | 0.43 |
| No | Ref | 1.45 (0.81, 2.59) | 1.44 (0.43, 4.78) | 0.18 |  |
| Yes | Ref | 1.11 (0.55, 2.26) | **2.34 (1.43, 3.83)** | 0.03 |  |
| Alcohol consumption |  |  |  |  | 0.18 |
| No | Ref | **1.84 (1.10, 3.06)** | **13.22 (3.13, 55.82)** | 0.05 |  |
| Yes | Ref | 1.27 (0.71, 2.26) | **2.64 (1.64, 4.27)** | < 0.001 |  |
| Obesity |  |  |  |  | 0.78 |
| No | Ref | 1.43 (0.80, 2.56) | **3.61 (1.65, 7.86)** | 0.004 |  |
| Yes | Ref | 1.16 (0.55, 2.44) | 1.91 (0.92, 3.96) | 0.82 |  |
| Diabetes mellitus |  |  |  |  | 0.81 |
| No | Ref | 1.36 (0.85, 2.18) | **3.07 (1.92, 4.90)** | 0.31 |  |
| Yes | Ref | 1.30 (0.30, 5.66) | 1.21 (0.37, 3.92) | 0.86 |  |
| Hypertension |  |  |  |  | 0.09 |
| No | Ref | **1.67 (1.06, 2.64)** | **3.83 (1.91, 7.70)** | < 0.001 |  |
| Yes | Ref | 1.14 (0.55, 2.39) | **1.91 (1.14, 3.19)** | 0.81 |  |

Bold denotes statistical significance at *P* < 0.05

Adjusted for age, gender, race, education level, marital status, PI, obesity, smoking status, alcohol consumption, diabetes mellitus except the subgroup variable.
